# A Nonlinear Cable Framework for Bidirectional Synaptic Plasticity

**DOI:** 10.1371/journal.pone.0102601

**Published:** 2014-08-22

**Authors:** Nicolangelo Iannella, Thomas Launey, Derek Abbott, Shigeru Tanaka

**Affiliations:** 1 Centre for Biomedical Engineering (CBME) and the School of Electrical & Electronic Engineering, The University of Adelaide SA, Adelaide, Australia; 2 Computational and Theoretical Neuroscience Laboratory, Institute for Telecommunications Research, University of South Australia, Mawson Lakes, South Australia, Australia; 3 Launey Research Unit, RIKEN, Brain Science Institute, Saitama, Japan; 4 Faculty of Electro-Communications, The University of Electro-Communications, Choju-shi, Tokyo, Japan; University of Sydney, Australia

## Abstract

Finding the rules underlying how axons of cortical neurons form neural circuits and modify their corresponding synaptic strength is the still subject of intense research. Experiments have shown that internal calcium concentration, and both the precise timing and temporal order of pre and postsynaptic action potentials, are important constituents governing whether the strength of a synapse located on the dendrite is increased or decreased. In particular, previous investigations focusing on spike timing-dependent plasticity (STDP) have typically observed an asymmetric temporal window governing changes in synaptic efficacy. Such a temporal window emphasizes that if a presynaptic spike, arriving at the synaptic terminal, precedes the generation of a postsynaptic action potential, then the synapse is potentiated; however if the temporal order is reversed, then depression occurs. Furthermore, recent experimental studies have now demonstrated that the temporal window also depends on the dendritic location of the synapse. Specifically, it was shown that in distal regions of the apical dendrite, the magnitude of potentiation was smaller and the window for depression was broader, when compared to observations from the proximal region of the dendrite. To date, the underlying mechanism(s) for such a distance-dependent effect is (are) currently unknown. Here, using the ionic cable theory framework in conjunction with the standard calcium based plasticity model, we show for the first time that such distance-dependent inhomogeneities in the temporal learning window for STDP can be largely explained by both the spatial and active properties of the dendrite.

## Introduction

Countless experiments have shown that neural activity can modify the efficacy or weight of a synaptic connection between two neurons. Specifically, neurons from the neocortex or hippocampus have often demonstrated that Long Term Potentiation (LTP) can be induced by using either high frequency stimulation or pairing low frequency stimulation with postsynaptic depolarization; while Long Term Depression (LTD) can be induced via low frequency stimulation only [1]–[5]. Experiments have further demonstrated that the attributes of cooperativity and associativity between synapses are usually required for the induction of either LTP or LTD [3], [6]–[9]. Furthermore, they have illustrated that distinct types of LTP and LTD have different domains of expression [10]–[12]. Nevertheless, several experiments conducted over the last decade have shown that the property of temporal specificity is also an important determinant controlling the direction of synaptic change [13]–[17]. Specifically, landmark experiments have demonstrated that the precise timing and temporal order between presynaptic input and postsynaptic action potential generation dictates whether a synapse's efficacy is potentiated or depressed [14]–[19]. From these studies, an asymmetric temporal window is found emphasizing that if a presynaptic input precedes the generation of a postsynaptic action potential, then the synapse is strengthened; but if the temporal order is reversed, then depression results. This type of plasticity is consequently called spike timing-dependent plasticity (STDP) [14], [18].

Recent experiments have now started to focus more towards understanding the underlying molecular apparatus responsible for synaptic change [12], [20]–[28]. Unfortunately, a complete description of the molecular signaling network underlying synaptic plasticity, based upon interactions between different signaling molecules, is far from complete. Nevertheless, many experiments have shown that changes in cellular calcium concentration are indispensable for plasticity to occur. Particularly, due to the nonlinear voltage dependence of the N-Methyl-D-Aspartate (NMDA) receptor, modest activation has been shown to induce LTD, while strong activation gives rise to LTP [4], [5], [29], [30]. Furthermore, the induction of either LTD or LTP is always subsequently correlated by either a modest or large change in intracellular calcium concentration in the synapse, due to calcium entry via NMDA receptor activation. However, NMDA receptor activation is not the only route where calcium entering the synapse can occur; activation of voltage dependent calcium channels or calcium release from internal stores can also lead to alterations in intracellular calcium concentration.

In parallel to these studies, previous investigations have established that neuronal morphology affects the spread of activity within dendrites in a nontrivial fashion. This naturally led to the proposition of how neuronal morphology affects the expression of synaptic plasticity. A previous experiment has illustrated how the profile of the STDP window varies along the apical dendrite of a layer 2/3 pyramidal cell [31]. Such spatial variations are typically characterized by a broadening of the LTD window and, to a lesser extent, a reduction in the maximal amplitude of the LTP window. Notably, this heterogeneity indicates that activity-dependent modifications may be location-dependent. This is a view further supported by the existence of a cooperative switch determining the sign of plasticity in distal dendrites of neocortical pyramidal cells [32].

The main theoretical effort toward understanding the biophysical basis of bidirectional synaptic plasticity including STDP has primarily focused on the development of a unified theoretical framework. This framework is based upon a simplified calcium-based model, in which a calcium transfer function approximates the final outcome of the molecular signaling network, and where calcium influx via NMDA receptors plays the pivotal role as the sole coincidence detector in determining whether synaptic efficacy is potentiated or depressed. However, current investigations have also begun to shed further insights that have put into question the validity of the single coincidence detector concept underlying plastic change [33]–[35]. In fact, several experiments have suggested that a second coincidence detector or signaling pathway underlies LTD and typically requires calcium influx via voltage dependent calcium channels, as predicted by a previous study [33]–[36]. Furthermore, activation of group I metabotropic glutamate receptors and IP_3_ gated release from internal stores have also been implicated, even though their role in STDP has only been investigated in somatosensory pyramidal cells [37], [38]. Whether IP_3_ gated calcium release from internal stores plays a role in neuronal cell types in different cortical regions has yet to be addressed. In light of these experimental findings, the currently accepted calcium-based model of bidirectional synaptic plasticity needs to be elaborated, especially to describe spatial variations in plasticity outcomes introduced by dendrites.

Efforts in developing a unified theoretical framework have been aimed at understanding the mechanisms underlying plasticity of synapses located on the dendrite. These efforts typically assume a simple yet phenomenological representation of the back propagating action potential, in which details of the spatial extent and active properties of the dendrite have been largely omitted. To date, the underlying mechanisms that can account for the location dependence of the STDP window are currently unknown and require further theoretical and experimental investigation. Efforts in developing a unified framework have, however, began focusing on developing more sophisticated cable based formulation to more accurately describe spatial influences; not just phenomenological approximations. Recently, a theoretical framework called *ionic cable theory* has been developed, which takes into account the voltage-dependent nature of ion channels and their physically discrete distribution throughout the membrane, and permits analytical solutions to be gained [39]–[42]. This framework has recently been extended to incorporate the spatial effects of reaction and diffusion of calcium [43], [44]. This extension allows an ideal starting point to investigate the effects of calcium-based synaptic plasticity in spatially extended dendrites, and observe any implications of the influence of neuronal morphology on plasticity outcomes.

In this paper, for the first time, we propose that the spatial and active properties of the dendrite play a dominant role underlying the location-dependence of the STDP window. We demonstrate how the properties of the dendrite contributes to location-dependent alterations in the STDP window by incorporating NMDA receptors into an extended version of the original ionic cable theory framework and unifying this construction with a biophysically inspired calcium-based model for bidirectional synaptic plasticity. Using this newly developed cable-based unified framework for bidirectional synaptic plasticity, we not only reproduce the location-dependence of STDP window, but also investigated pairing frequency effects as a function of location along a dendrite. Our study predicts a novel increase in the repetition pairing frequency required to switch from LTD to LTP for negative post-pre pairings and further suggests a nontrivial activity dependent multi-dimensional parameter space for inducing synaptic change along dendrites.

## Results

### Cable-based framework application of calcium based plasticity models of STDP

The ionic cable framework is applied to study calcium based synaptic plasticity in a section of dendrite. Here, the calcium dependent plasticity (CaDP) model, originally proposed by [45], is extended to take into account the spatial nature of the dendrite, and where alterations to synaptic strength are driven by calcium entry through voltage-dependent calcium channels and NMDA receptors. A complete formulation of this framework and its application to CaDP is provided in the materials and methods section. In the original model by Shouval et al. [45], plastic change occurs through a single point of association that relies upon the interaction between glutamatergic NMDA receptor activation and strong depolarization of the postsynaptic membrane, where NMDA acts as a coincidence detector of pre- and postsynaptic activity. This model explicitly implements the calcium control hypothesis where moderate levels of calcium above some baseline leads to LTD while high levels of calcium gives rise to LTP. Furthermore no location-dependent variation in the parameters of the plasticity rule was used throughout this study in order not to obscure outcomes due to the dendrite with other sources. Put simply, one wants to investigate the effect of the dendrite has on plasticity outcomes without any additional location-dependent sources.

**Figure 1:**
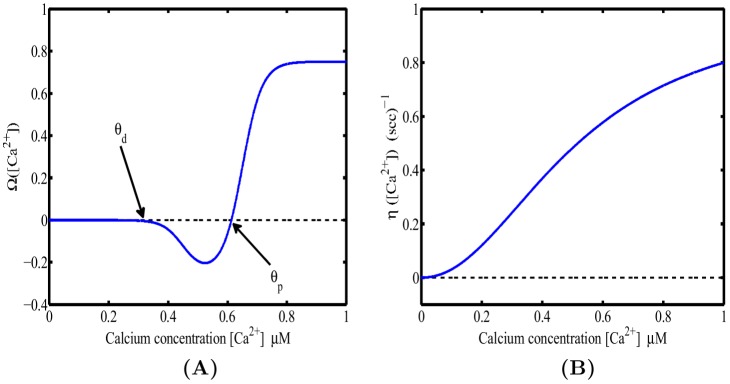
(**A**): The calcium dependent function 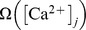 implements the calcium control hypothesis, when 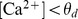 no change in the synaptic weight occurs, for 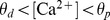 synaptic depression (LTD) occurs, and for 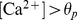 synaptic weights are increased (LTP) (parameters used were 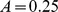, 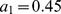, 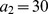, 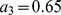, 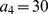. **B**: Calcium dependent learning rate 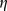 (adapted from [45]). Parameters were 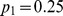, 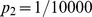, 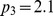, 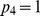.

Recalling that changes to synaptic strength in the original CaDP model proposed by Shouval [45] is given by the set of equations below (Eqn. 1) but restated here for convenience, 
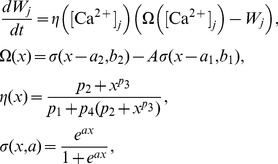
(1)where 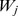 denotes the strength of synapse 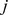, 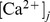 is the peak calcium concentration at that synapse, 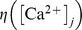 is a calcium dependent learning rate, and 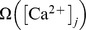 determines the sign of synaptic change as a function of calcium concentration 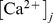 in synapse 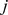. The calcium dependent functions 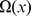 and 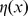 are illustrated in Figure 1A and 1B, respectively. The weight change to a single presynaptic and postsynaptic pairing is proportional to
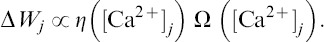
(2)


For this example, a single hotspot of NMDA receptors along with a standard set of ion channels, whose descriptions are based on the Hodgkin-Huxley formalism, were used. This set was comprised of sodium, the delayed rectifier and transient potassium, and both low voltage activated (LVA) T-type and high voltage activated (HVA) L-type calcium channels. As described in the materials and methods section, a set of reaction-diffusion equations can be rewritten into a corresponding system of cable equations for the general case where multiple NMDA hotspots are present is given by the following set of equations, noting that we have already transformed to dimensionless variables where 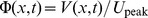, (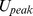 is the peak value of the membrane potential) represents dimensionless voltage, and 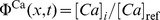 represents a dimensionless concentration, 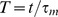, 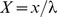, 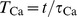 and 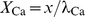 are the dimensionless space and time variables for the system of cable equations (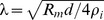, 
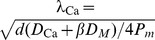
, 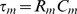, 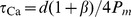, and 
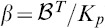
). Note that for any dimensionless position on the electrotonic cable and its corresponding location on the chemicotonic cable equation, these values will differ however one must keep in mind that these differing values represent the same position 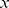 in physical space. We present the corresponding system of cable equations for dimensionless voltage 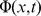 and correspondingly for dimensionless calcium concentration 
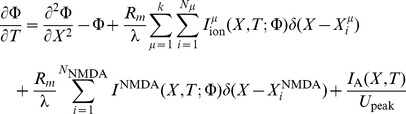
(3)

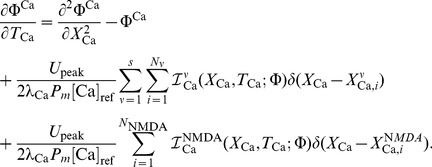
(4)


For the illustrative purposes of this study, we assume a semi-infinite long cable of constant diameter where the initial conditions of the entire system is at rest, along with a nonhomogeneous Dirichlet boundary condition at 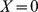, which implements a nonlinear current clamp with the shape of an action potential, i.e. 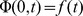, while for impulsive inputs occurring along the cable, we assume that the current clamp is set to zero hence the corresponding boundary condition for these inputs is equivalent to the killed end condition 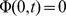. The summation appearing in Eqn. (3) represents the sum over all voltage dependent ionic current sources and is given by 
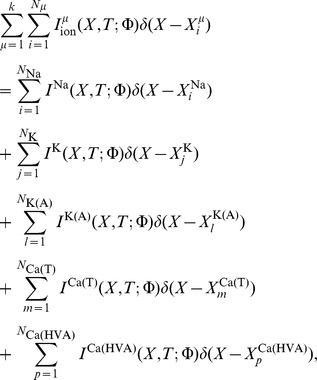



while the corresponding term in Eqn. (4) indicates the sum over all voltage gated calcium channels. 
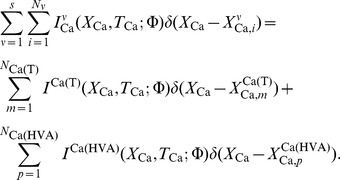



When presynaptic stimulation occurs at time 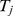, the NMDA receptor current and the corresponding calcium current through this receptor are given by 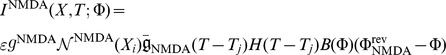


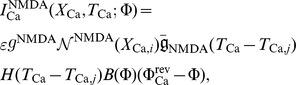
where 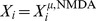 represents the electrotonic positions of the respective 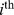 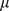 channel and NMDA receptor hotspots. Similarly, 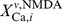 denotes the corresponding positions of calcium currents generated by the 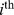 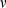-type calcium channel and NMDA receptor hotspots, respectively. Here, 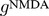 is the single channel conductance of NMDA, 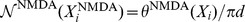 corresponds to the number of NMDA receptors per unit length (cm^−1^) and 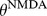 represents the number of NMDA receptors in the 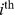 NMDA receptor hotspot, 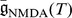 is the conductance change of the receptor, 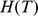 denotes the Heaviside step function, 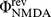 and 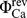 are the respective reversal potentials for NMDA and calcium, and magnesium block is represented by a nonlinear voltage dependent function 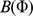 [46] given by 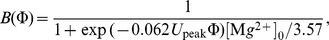
where 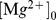 is the extracellular magnesium concentration. Action potentials from hippocampal and somatosensory pyramidal cells [47], [48] typically possess an after depolarizing tail and have been shown to participate in synaptic plasticity due to increased calcium influx through NMDA receptors and voltage gated calcium channels (VGCC) [47], [49]–[52]. In order to model these action potentials, the stimulation to the cable at 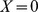 was accordingly modified to, 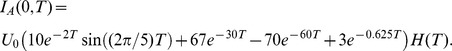



Calcium profiles resulting from the coincidence between presynaptic activation of an NMDA receptor hotspot and action potential propagation into a dendritic section are calculated by recasting Eqns. (3) and (4) into their corresponding system of integral equations, as described in the materials and methods section. Membrane depolarization along the dendritic section is evaluated to first order in the perturbation series expansion, which is consequently used to calculate the resulting calcium profiles from Eqn. (14). The resulting calcium profiles can then be used to evaluate the change in synaptic weight. The parameters used in the following sets of simulations were 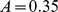, 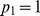, 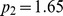, 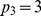, 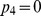, 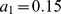, 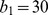, 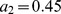, and 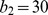, unless stated otherwise.

#### Dendritic morphology as an important contributor to the spatial dependence of STDP

In this illustrative example, we show how the spatial nature of the dendrite, along with its distribution of ion channels, can play an influential role in shaping the resultant STDP window obtained by calcium-based plasticity. Specifically, we will illustrate how the mere presence of an elongated (spatially extended) section of dendrite (a simple cable representation) can reproduce both the elongation and increase in the decay time constant of the LTD portion of the STDP window, as seen in experiments by [31]. We will further investigate how variations to the diameter of the dendrite, and alterations to the ion channel densities for sodium, high and low voltage activated (L-type and T-type) calcium channels. For all these cases, a single NMDA receptor hotspot is used to determined weight changes from two different locations (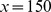 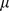
m and 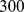 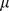
m) along the dendrite. The STDP window is determined by initially calculating the resultant calcium concentration profile at the location of the NMDA receptor hotspot, and then using the maximally obtained calcium concentration, changes to the synaptic weight are readily calculable where one can construct a temporal window for synaptic change at the preferred/chosen location containing the NMDA receptor.

**Figure 2:**
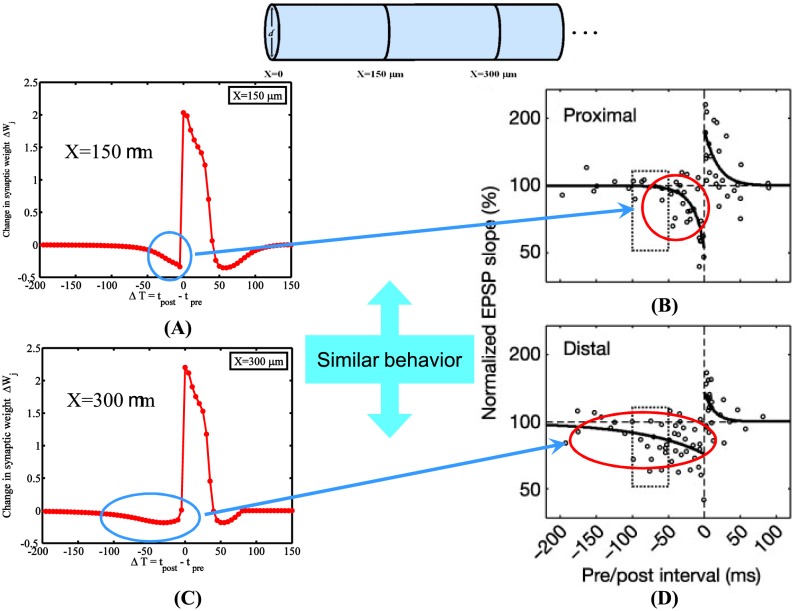
Resulting STDP learning windows taken from two different positions along the cable. In (**A**) and (**C**), note how the illustrated plasticity outcomes for a single pre- and postsynaptic pairing using Eqn. (2), derived from the calcium control hypothesis using calcium based plasticity rule, changes as a function of distance. In particular, comparing (**A**) to (**C**) note the increase in the time constant of the LTD portion of the STDP window when the position from the source of spike initiation is increased. This behavior mimics the location-dependent nature of the STDP window as observed in (**C**) and (**D**) as presented in previous physiological experiment [31]. Parameters used were 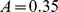, 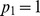, 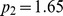, 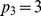, 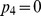, 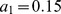, 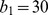, 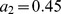, and 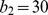. (Figs 2B and 2D were adapted from [31]).

Figure 2 illustrates plasticity outcomes for a single pre- and postsynaptic pairing derived from Shouval's CaDP model [45] taken from two different (electrotonic) positions, 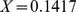 and 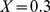 (corresponding to 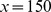 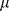
m and 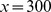 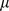
m, respectively), along the cable. In order to calculate the STDP learning window using the calcium dependent plasticity (CaDP) model presented in [45], peak calcium concentrations were normalized prior to determining the size and magnitude of plastic change. We can clearly see in Figure 2 that the cable-based model of calcium-dependent plasticity, successfully reproduced the location-dependent nature of the STDP window, as previously observed in experiments [31]. To date, no other model has been able to illustrate this using a unified framework based upon cable equations. We find that the mechanism behind the location dependence of the STDP window is not only driven by biochemical cascades interacting with calcium influx into the cystol (whether it is through the plasma membrane via voltage dependent calcium channels or NMDA receptors; or via calcium-dependent calcium release), but also there is a strong dependence on the spatial nature of the dendrite that plays an important role in shaping electrical activity (mainly via voltage attenuation). For this model, however, there is a region of the temporal learning window that is partially supported by experimental data [53]. Note that even though the STDP window derived from Eqn. (2) in Figure 2A and 2C has an LTD region at positive pre-postsynaptic timings, most experimentally observed STDP windows have not shown this secondary LTD component, except for in a few specialized investigations [33], [34]. These potential short-comings arise since coincidence detection of calcium through NMDA or VGCC is a directly proportional measure of plastic change, while the pre-post form of LTD arises since normalized calcium concentrations for timing intervals 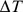 between 40 to 100 (msec), are at intermediate values between LTP and where no change occurs, falling into the region where 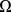 gives rise to LTD. Experimental evidence supporting pre-post LTD has been controversial since most experiments using CA3-CA1 synapses have not observed it, despite few experiments illustrating its existence, but these studies have emphasized that its probable cause may be attributed to inhibition within slice preparations [53], [54] or maybe due to the biochemical nature of calcium signaling cascades [33], [34]. To date, this specific controversy has yet to be fully resolved.

**Figure 3:**
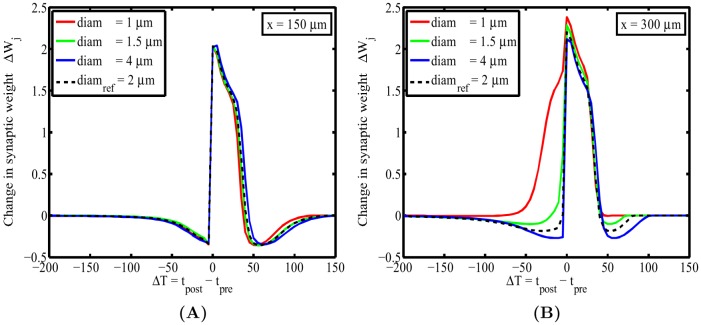
Simple changes to morphology by changing the diameter results in visible changes to the STDP window taken from two different positions along the cable. (**A**) For the first location at 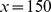 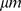, it seems that the influence of varying cable diameter is negligible but for (**B**) the second position at 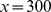 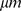, we observe more prominent changes to the STDP window, in particular the disappearance of LTD portion of the window for decreasing diameters. This is mainly caused through larger normalized calcium concentration values that are used to calculate synaptic weight changes.

Numerous studies in the past have shown that changes to spatial morphology lead to altered firing properties [55]–[58] that inherently result in different spatiotemporal profiles of calcium along dendrites. Such changes in calcium profiles are capable of leading to changes in calcium-dependent plasticity outcomes. This poses the question of how simple alterations to morphology, such as changes in diameter, modify the shape of the STDP window. Figure 3 illustrates how changes in diameter alter the location dependence of the STDP window in a nontrivial fashion, in particular for the case when the diameter is decreased. The LTD component of the STDP window is dramatically reduced for the more distal location along the cable. This is caused by having larger normalized calcium values that drive synaptic change at this more distant position along the cable.

#### Neuronal excitability as an additional contributor to the spatial dependence of STDP

**Figure 4:**
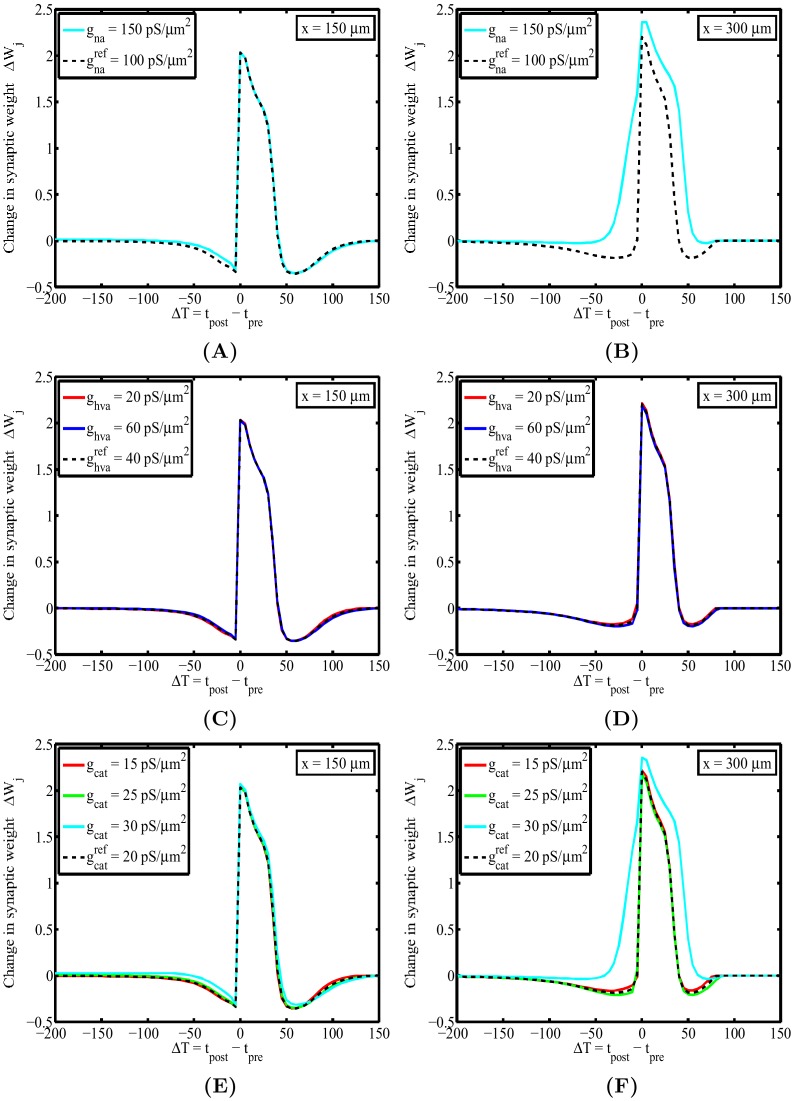
Changes in excitability through changes in ion channel hotspot density can change the location dependence of the STDP window to different degrees. (**A**) shows how increasing the density of sodium channels by 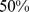 had little change on the profile of the STDP window for the first location at 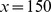 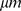, but for (**B**) the second location at 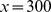 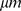 there is an evident change in the STDP window where the LTD contribution has all but vanished for the increased sodium channel density. This is attributed to the increased calcium influx into the location of the dendrite. (**C**) and (**D**) illustrate that varying the density of high-voltage activated had little impact on both the profiles and the spatial dependence of the STDP window. (**E**) and (**F**) shows that increasing the density of low-voltage activated calcium channels by 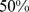 gave rise to similar changes in the profile (and spatial dependence) of the STDP window at the two pre-specified locations, however for smaller variations few differences were observed.

Changes to neuronal excitability can lead to dramatic changes in the response of a neuron and the influx of calcium locally in the dendrite. These changes will lead to altered plasticity outcomes, hence it is natural to ask how altering the excitability of a section of dendrite through changes in the density of ion channels for specific types of hotspots alters the location dependence of the STDP. Here, we directly observe the impact of altering the densities of sodium, low voltage activated (T-type) and high voltage activated (L-type) calcium channels on the spatial dependence of the STDP window. Figure 4 illustrate how changing these specified channel densities alters the STDP window. Figures 4A and 4B show how increasing the density of sodium channels from 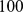 pS/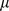
m^2^ to 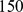 pS/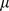
m^2^ (a 50% increase) changes the spatial dependence of the STDP window. For the more proximal position, the increase in sodium channel density had little impact on the window, however for the distal location 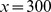 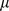
m the STDP was altered to the extent that the LTD contributions have disappeared. This is explained since the higher density of sodium channels leads to larger dendritic depolarizations that give rise to higher levels of calcium, which in turn affect the STDP window through an increase in the LTP contribution. Figures 4C and 4D illustrate how changing the channel density of high-voltage activated (L-type) calcium by 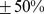 led to negligible changes in the spatial dependence of the STDP window, indicating that there was little contribution to the total amount of calcium when compared to other calcium influx through T-type calcium channels and NMDA receptors. Finally, Figures 4E and 4F show how increasing the density of low-voltage activated (T-type) calcium channels by 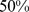 leads to changes in the spatial dependence of the STDP window similar to those seen for increasing the density of sodium channels, namely the loss of and notable LTD component due to increased calcium influx, while smaller increases and decreases resulted in little change.

#### Spatial dependence of STDP pairing frequency effects

The spatial influence of the dendrite is not just limited to altering the STDP learning window; it also leads to the prediction that spatially dependent changes to the outcomes of pairing frequency effects should also be potentially observable. Here, we show the outcomes of calcium dependent plasticity when a single hotspot of NMDA receptors is positioned at two different (electrotonic) locations, 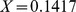 and 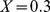 and along the dendrite. We illustrate how the intrinsic spatial nature of the dendrite naturally gives rise to a location dependence for pairing frequency effects. In order to calculate the calcium driven changes we simulate the STDP pairing frequency protocol. Here, the cable is subject to action potential generation and presynaptic stimulation, where a postsynaptic spike is paired with a pre-synaptic input, which defines a pairing event with a time difference 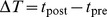 of 10 msec. This pattern of stimulation is repeated sixty times, where the timing difference between pairing events is initially set at 1 Hz. The simulation is then repeated with the same stimulation sequence of sixty pairing however for these repeated simulations, the pair presentation frequency (the time between presentation of pairing events) is increased from 1 Hz to 60 Hz. We then change the time difference between pairing events to 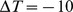 msec and repeat the entire process again.

**Figure 5:**
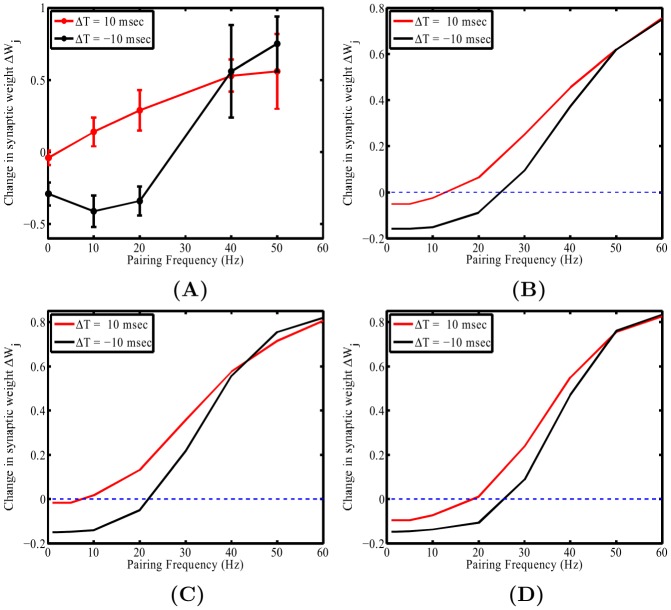
The outcomes of pairing frequency effects taken from Sjöström's original experiment [59] and the simulation. In **A**, we present the original data by Sjöström [59] for comparison with our simulation results. In **B**, we display the outcome of Shouval's calcium based plasticity rule where changes are driven by normalized peak calcium. Note how the resulting plasticity profiles for 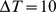 and 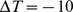 as a function of frequency lack a notable crossover point at 40 Hz. In **C** and **D**, again CaDP is used but this is driven by a modified calcium-dependent variable where the ratio between the peak calcium and the maximal integral over time of the calcium concentration profile is used to drive plastic change. In **C** and **D**, one can observe distance dependent changes in the plasticity outcomes for the pairing frequency protocol (as explained in text). In **C**, we see that the 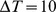 msec curve gives rise to only LTP, while for the 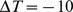 msec there is an initial region that gives rise to LTD but then at a particular frequency there is a switch from LTD to LTP. Furthermore, note that the two curves crossover at 42 Hz. This is similar to Sjöström's experimental findings in **A**. In **D**, one can see a rightward shift of the 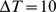 msec curve where it inherits a small LTD component, however for the 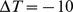 msec data there is a notable rightward shift of the LTD region leading to increase in frequency when LTD switches to LTP.

Figure 5A illustrates the original experimental results adapted from Sjöström et al. (2001) [59], and Figures 5C and 5D present the plasticity outcomes for the pairing frequency protocol calculated using Shouval's CaDP model [45] for two different (electrotonic) positions, 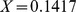 and 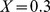, along the cable. In Figure 5B we find that normalizing the peak calcium concentration gave rise to results that lacked the noticeable crossover frequency between the 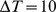 and 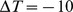 profiles at approximately 40 Hz. To obtain a better match to the experimental profiles, we find that using the ratio between the peak calcium concentration and the maximum of the integral of the calcium concentration profile over time gives rise to plasticity changes in Figures 5C and 5D that are in better qualitative agreement with the experimental results of Sjöström et al. [59] displaying a crossover at approximately 43 Hz. Our motivation for this naive yet convenient modification, is that synaptic change in plasticity experiments is usually seen after multiple stimulus presentations and rarely after a single stimulus event. The main biophysical reason for notable synaptic change occurring after multiple (stimulus) presentations is that changes in AMPA receptor numbers in the post synaptic density typically require some functional form of temporal integration of calcium activity into second messenger and other biochemical signaling pathways, which ultimately leads to either removal or inclusion of AMPA receptors. By this we mean that changes in AMPA receptor density are functionally dependent on the calcium activity integrated over time. The parameters used in the repetition frequency protocol were 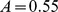, 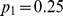, 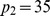, 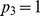, 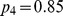, 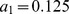, 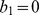, 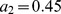, and 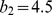.

Using this modification to CaDP, we can clearly see that the effect of dendritic spatial extent has, when comparing the resulting data from 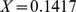 to 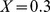. We observed spatially dependent differences between the data from 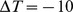 msec and 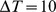 msec. Specifically, we find that there is a rightward shift in both curves, in which the 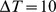 msec curve developed a small region where LTD is produced and the 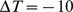 msec curve illustrated a clear increase in the frequency where LTD switches to LTP. In addition to the known complexity of the spine biochemistry, the work of this paper suggests a novel prediction where the increase in repetition frequency leading to the switching from LTD to LTP occurs as a function of increasing distance from the soma. This prediction can be verified with current electrophysiological setups and techniques. It has been known that a complex interplay between dendritic structure, membrane excitability, and synaptic inputs determines how a neuron will respond to synaptic stimulation [58], [60]–[61]. In turn, this suggests that dendritic morphology, membrane excitability, and the repetition frequency play important roles in determining, at any particular location on the dendrite, whether plasticity leads to LTD or LTP, in particular the unique frequency where LTD switches to LTP and illustrates that such a unique frequency is not constant but varies in a location-dependent fashion. Finally, the results presented here and as suggested in [62], indicate that this interplay can be viewed in establishing a nontrivial, spatially dependent, but activity driven higher dimensional parameter space determining the conditions such as times, locations, and magnitude of synaptic change along the dendrite.

## Discussion

Passive or linear cable theory has provided many useful insights in how neurons function, particularly it was instrumental in providing the first solid theoretical framework for understanding how synaptic integration occurs across the dendrite [58], [60], [61], and specifically showed how depolarization caused by spatially separated synaptic inputs can interact and spread throughout a dendritic structure. In order to capture the nonlinear nature of the membrane, attempts at formulating a nonlinear version of cable theory has been developed via the so-called theory of N-dendrites by [63], in order to study dendritic bistability in a semi-quantitative fashion. Only recently have there been some attempts to find analytical solutions to nonlinear cable equations [39], [40], [42]–[44], [64]–[66]. These attempts have taken advantage of one important fact that ionic channels are pores embedded in the neuronal membrane [67] whose distribution is not continuous but discrete [68], [69]. This consequently permits ionic channels to be treated as discrete current sources, and which led to the development of *ionic cable theory* [39], [40], [42]. In recent studies, this approach was expanded to include calcium diffusion and buffer systems [43], [44].

This extended framework has previously been used to investigate the propagation of both an action potential and a spike train in a model cable with calcium dynamics, while subject to the influence of various ion channels [44]. Here, this cable-based framework was applied to study the calcium control hypothesis based upon the calcium based model of STDP proposed by [45]. Note that, NMDA receptors were included into this new framework and calcium transients were calculated subject to the precise timing between a BPAP (possessing a slow after-depolarizing tail) and presynaptic activation of a single NMDA receptor hotspot at two different spatial locations along a cable. Adopting Shouval's original CaDP model [45], the resulting STDP learning window had both favorable and unfavorable features. Interestingly, the time constant for the post-pre LTD component (at negative ISIs) of the window increased as a function of distance, consistent with a recent experimental study [31]. However, the appearance of a secondary causal pre-post LTD window, is not fully supported by past experiments. Evidence supporting the appearance of this secondary LTD window is controversial since few studies have reported it, despite concluding that its probable cause was inhibition within the slice [53], [54], most experiments do not see this pre-post form of LTD. This is further confounded by the fact that pre-post LTD has, to date, only been observed in hippocampal CA3-CA1 synapses and recent experiments have shown that STDP at these synapses can display different learning windows depending on the physiological state and suggested that two rules, one for LTD and the other for LTP, may govern plasticity outcomes [33], [34]. The appearance of this secondary LTD window and its underlying (molecular) mechanism(s) have yet to be fully resolved or understood.

For more than two decades there has been a clear trend for using computational platforms such as NEURON or Genesis (now rebranded as Moose) that implements numerical techniques for solving problems involving spatially extended neurons. The main reason for the dominance of numerical as opposed to analytical techniques has been the widespread availability of high performance computers that have allowed analysis of many different problems. It is well known fact that for many problems, a numerical solution is the readily achievable while obtaining an analytical closed-form solution is not possible. Then there are cases where the solution can be calculated analytically, but may be more convenient to find the solution numerically. From a historical perspective, the ionic cable-based model presented here may seem a backward technological step; however the main motivation for this model was not to increase computational speed, but rather to provide a framework to better understand how the underlying complex dynamics associated with membrane voltage and calcium dynamics may impact the outcomes of synaptic plasticity. Moreover, the model and framework provides a unified methodology that makes it easier to understand the dynamical outcomes of plasticity and any associated manipulations. Furthermore, due to an inherent equivalence, the model can draw from the vast knowledge and experience of the extensively studied electrical cable equation, and further provides important insights and interpretations about the functional nature of the calcium reaction-diffusion subsystem and the potential outcomes of calcium based synaptic plasticity.

Our study highlights how the interaction between electrical and chemical systems can be combined in a way that permits useful insights on how the spatial extent of the dendrite can influence plasticity outcomes. In particular, we found our framework useful in illustrating the importance of spatially extended dendrites on the outcomes of various plasticity inducing paradigms. Furthermore, we emphasize the view that dendrites are not just considered a signaling medium, but their spatial morphology plays an important role in shaping distance-dependent influences on the outcomes of synaptic plasticity, which consequently shapes the spatiotemporal pattern of synaptic inputs onto the dendritic trees of individual cortical neurons.

### Limitations of the Study

Our ionic cable theory framework has provided valuable insights and potential experimentally viable predictions. Despite its usefulness, there are potential improvements that can be addressed in future work. The most obvious improvement is not to artificially voltage clamp the boundary of the cable to the shape of a spike, but allow it to be generated naturally by adding an active somatic compartment. Future work may consider development of an improved description of the calcium subsystem that does not have the requirement for the fast buffer approximation. This will rely on using special Green's function techniques [70] to describe the dynamics of the calcium subsystem and its interactions with calcium buffers of varying affinities. Another novel improvement is envisaged by employing singular perturbation theory to deal with the different dynamical time scales between voltage and calcium/biochemical reaction and diffusion. An additional improvement is to introduce dendritic spines so that one can gain a better understanding how the morphology of simple spines can influence voltage and calcium dynamics both within the spine apparatus and the dendrite. A final improvement that can be employed is the application of singular perturbation theory (as opposed to regular perturbation theory) to obtain more accurate descriptions of the coupled voltage-calcium dynamics, in particular the underlying nonlinear coupling between voltage, calcium, and mobile buffers. Implementing these improvements to our current ionic cable theory framework is viewed to provide a deeper dynamical understanding of signal transmission and integration in the biochemical environment within dendrites.

## Conclusions

This current study illustrates how the interdependence between electrical and chemical cables can provide useful insights into distance-dependent plasticity phenomena in spatially extended dendrites. Further extensions to this framework are currently being pursued, such as the incorporation of dendritic spines into the framework and more sophisticated calcium signaling biochemistry. Such new additions will allow one to study the interplay between synapse location, synaptic plasticity, and the details of how spines and dendrites impacts STDP outcomes. The potential usefulness of this extended framework is not only to study calcium-based models of synaptic plasticity, but also to gain valuable insights how altering ion channel current distributions and membrane excitability may influence plasticity outcomes or put simply how altering excitability impacts plasticity in dendrites. As a final note, the complex interplay between synaptic plasticity, spatial morphology, neuronal excitability, and input activity play critical roles in setting up the sites, moments, degree and direction of changes to spatially distributed synapses located in neuronal dendrites.

## Materials and Methods

### From Reaction-Diffusion Dynamics to System of Cable Equations

**Figure 6:**
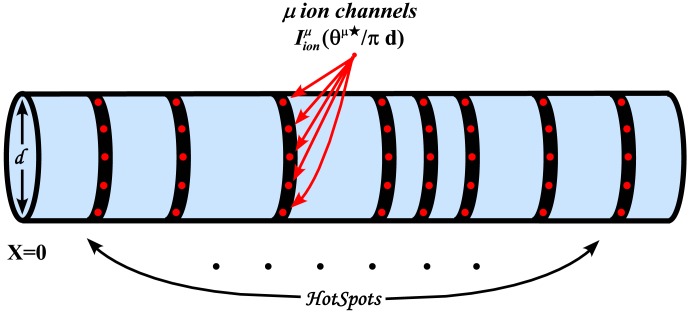
A schematic illustration of a dendritic cable studded with 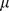 ion channel hotspots. A single hotspot is represented as a black band and denotes an infinitesimal area on the cable, containing 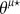 ion channels of type 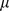 where single ion channels are represented as red spots.

As previously described in [43], [44], let us consider a model neuron as a continuous cable, whose voltage at space point 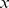 (cm) and time 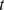 (msec) is denoted by 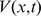 (mV). Furthermore, for simplicity, calcium diffuses through the cable in the presence of calcium pumps and a single buffer type. Simulations which include the effects of calcium exchangers are left as an exercise for the interested reader. As presented in [43], [44], voltage and/or calcium dependent ion channels are distributed along the cable at discrete loci forming hotspots as shown in Figure 6. These hotspots contain a cluster of channels which act as point current sources. The system of reaction-diffusion equations to be considered is then given by, 
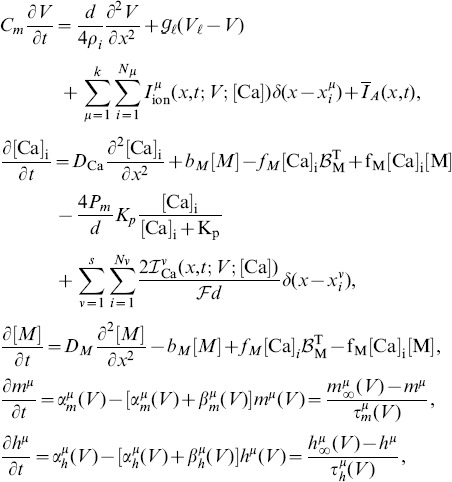
(5)where 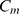 is the membrane capacitance per unit area (F/cm^2^), 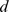 is the diameter of the cable, 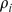 is the internal cytoplasmic resistivity (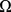 cm), 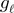 is the leak conductance (S/cm^2^) and 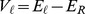 is the reversal potential (mV) where 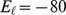 mV is the equilibrium potential for the leak current and 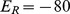 mV is the resting membrane potential, respectively. Note that 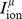 is the ion current density per unit length (mA/cm) (which can either be voltage dependent, calcium dependent or both) for a single type of channel, denoted by 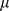. Here, 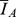 is an applied current density per unit area (mA/cm^2^) representing either synaptic input or an external current, and 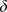 is the Dirac delta function (cm^−1^), where 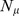 and 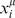, are the numbers and positions of hotspots for each respective channel 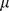. The summations over channel types 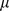 and hotspot loci 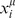 represents the sum over all voltage dependent current densities.

The internal calcium and buffer concentrations (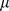
M) at position 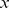 and time 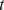 are correspondingly denoted by 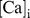 and 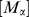, and 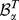 denotes the total concentration of buffer 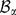. The corresponding diffusion coefficients are given by 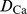 and 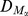 (in units of 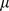
m^2^/msec). Calcium ions are extruded from the cable via a high affinity calcium pump where 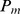 is the membrane pump parameter (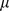
m /msec), 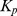 (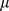
M) the pump dissociation constant and the factor 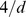 is the surface area-to-volume ratio for a cylinder of diameter 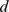. Calcium entry into the cable is carried by the calcium current density 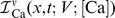 flowing through each specific voltage and calcium dependent calcium channel 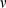. The ratio 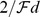 ensures that the current density per unit length is converted into a concentration gradient, where 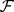 is Faraday's constant (C/mol). The summations over calcium channel type 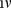 and their corresponding hotspot locations 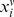 represents the total calcium influx entering the cable through all calcium channels.

The steady state activation variable 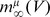 and its associated time constant 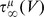 for channel 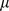 are given by 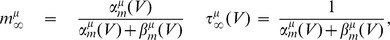
where 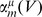 and 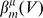 are the voltage dependent forward and backward reaction rates. Similar expressions apply for the 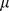 channel steady state inactivation variable 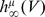 and corresponding time constant 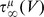.

As described in [43], the calcium system can be linearized using the rapid buffer approximation. This approximation allows the system of reaction-diffusion equations can be reduced to the following set of equations, 
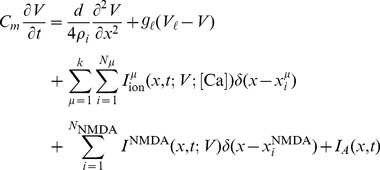
(6)

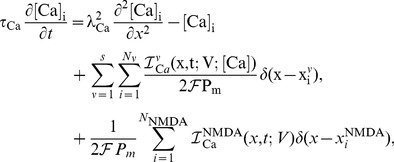
(7)where the applied current density per unit area 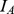 represents an externally applied input, 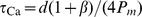, 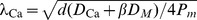 and 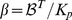.

The above system of cable equations, given by Eqns. (6) and (7), can be rewritten in terms of dimensionless quantities. This is achieved by introducing the membrane resistivity 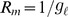 (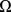 cm^2^), the dimensionless membrane potential 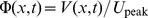, where 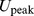 is the peak value of the membrane potential, a dimensionless concentration 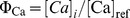, and changing to dimensionless space 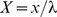 and time 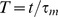 variables in Equation (6), where 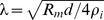 (cm) is the space constant and 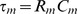 (msec) is the membrane time constant. While for the chemical cable equation Eqn. (7), the dimensionless space and time variables are defined as 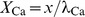 and 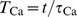, where 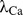 and 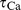 are the corresponding space and time constants. By using the following property of the Dirac delta function, 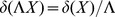, the resulting dimensionless system of cable equations is given by 
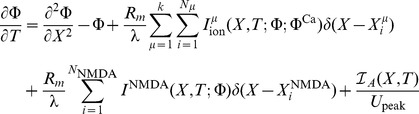
(8)

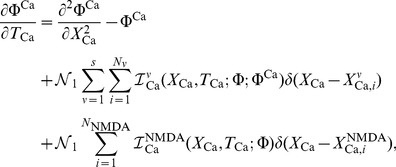
(9)where 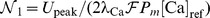, the applied current is 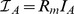 and 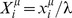 and 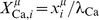 denotes the corresponding location of the 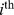 hotspot for channel 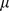 in the cable and chemical cable, respectively. The summation appearing in Eqns. (8) 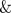 (9), represents the sum over all voltage dependent ionic current sources. Furthermore, we emphasize that although the electrotonic location of hotspots occurring in the dimensionless cable and those chemicotonic locations in the dimensionless chemical counterpart differ, they each represent the same physical location in space along the cable.

### Reformulation as a system of nonlinear integral equations

In order to find analytical solutions to the system of cable equations defined by Eqns. (8) and (9), ion channel descriptions are required. In most studies, the description of ion channels are typically based upon either the Hodgkin-Huxley (HH) or the Goldman-Hodgkin-Katz (GHK) formalism. Thus, the ionic current density generated at 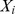 by channel 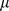 can be generally defined as 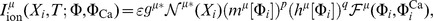
where 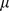 denotes the ion channel type, 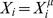 corresponds to the electrotonic position of the 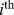 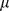 channel hotspot, 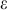 is a parameter scaling the “whole-cell” macroscopic transmembrane current density into spatially discrete clusters of ion channels, 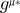 is the single channel conductance, 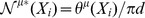 denotes the number of channels per unit length, with units of (cm)^−1^, and 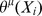 represents the number of channels in the 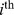 hotspot of channel 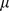, 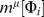 and 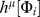 respectively represent the activation and inactivation variables of channel 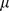, 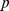 and 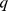 are exponents, and 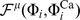 represents the voltage and/or calcium current dependence of channel 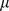. The current density generated by a single NMDA receptor hotspot at position 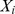 and its corresponding location in the chemicotonic cable 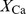


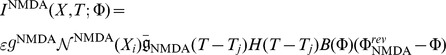


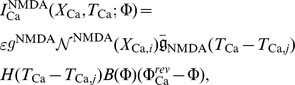
where 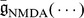 is the conductance change, 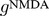 is the single channel conductance of NMDA, 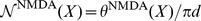 represents the number of NMDA receptors per unit length (cm^−1^) and 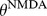 is the number of NMDA receptors in the 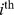 NMDA receptor hotspot 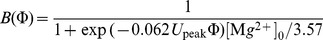
and 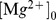 is the extracellular magnesium concentration.

As described in [43], by considering these ion channel hotspots act as point current sources, this permits the system of cable equations, Eqns. (8) and (9) to be recast into the following system of nonlinear Volterra integral equations, 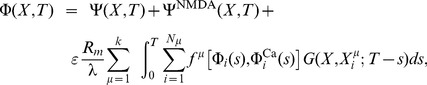
(10)

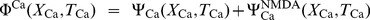
(11)

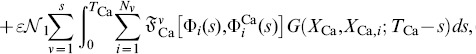
(12)where 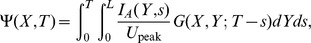


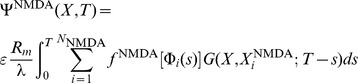


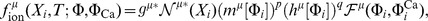


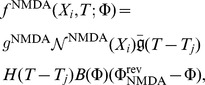


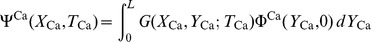


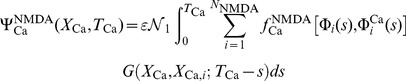
and 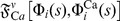 represents a voltage gated calcium channel with an explicit calcium dependence. The reader should keep in mind that although channel descriptions have been written with an explicit calcium dependence 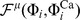, not all channels depend on calcium. This notation simply allows for a compact representation and does not mean every channel is calcium dependent; this point should not be confused.

For the case where an explicit calcium dependence is assumed or required, analytical expressions can be obtained through a regular perturbative expansion in terms of both 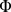 and 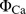. Such an expansion leads to expressions involving derivative cross terms with respect to dimensionless voltage 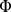 and dimensionless calcium 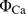; for an example of such an expansion see [44] for further details. In the past, many theoretical studies, such as those by [71], [ 72], have used ion channels including voltage-gated calcium channels with no explicit calcium dependence. In this paper, similar to these previous studies, we only consider voltage-dependent calcium channels with no explicit calcium dependence.

For such ion channels, the resulting system of cable equations is given by
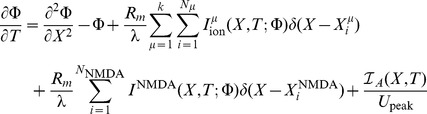


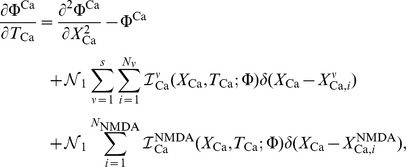
leading to the following analytical expressions for each of the perturbative terms appearing in the expansion of 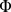 (see [43], [44] for further details), 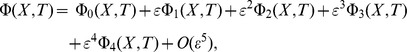
where 
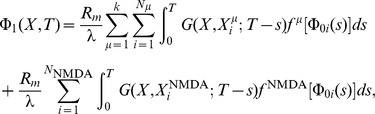


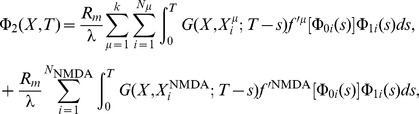


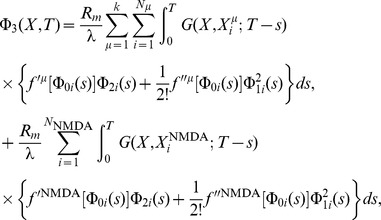


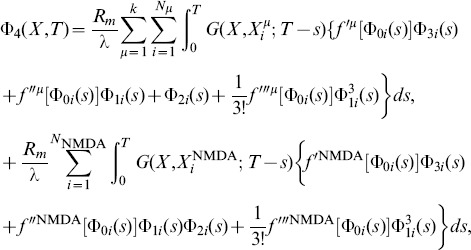
(13)and
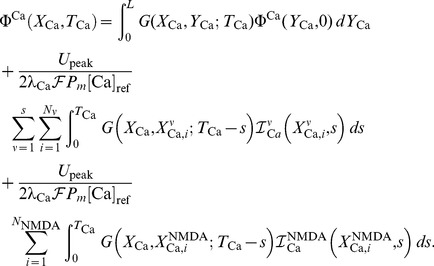
(14)Note that the integral expressions represent convolutions between the Green's function and various functional forms involving zero, first, and higher order terms of the ionic current and voltage contributions calculated via a regular perturbation expansion.

## Supporting Information

Information S1 (PDF)
