# Supplementary material for: A Nonlinear Cable Framework for Bidirectional Synaptic Plasticity
Source: PLoS One. 2014 Aug 22;9(8):e102601. doi: 10.1371/journal.pone.0102601 (PMC4141722; doi:10.1371/journal.pone.0102601)
Supplement: Information S1 — (PDF) [file pone.0102601.s001.pdf]

Supplementary Information - A nonlinear cable framework for bidirectional synaptic plasticity

Nicolangelo Iannella[1,2,3]*, Thomas Launey[3], Derek Abbott[1], Shigeru Tanaka[4]

[1] Centre for Biomedical Engineering (CBME) and the School of Electrical and Electronic Engineering
The University of Adelaide, SA 5005, Australia

[2] Computational and Theoretical Neuroscience Laboratory, Institute for Telecommunications Research,
University of South Australia, Mawson Lakes, South Australia, Australia

[3] Launey Research Unit for Molecular Neurocybernetics, RIKEN, Brain Science Institute, Japan

[4] Faculty of Electro-Communications, The University of Electro-Communications, Japan

July 29, 2014

Passive cable solutions and channel descriptions

The simulations required the following integral for $\Phi_0(X,T)$, representing the solution to the linear cable equation, to be evaluated:

$$\Phi_0(X,T) \;=\; \int_0^T \frac{I_A(0,s)}{U_{\text{peak}}} G(X,0;T-s)\,dY\,ds,$$

where $I_A(0,T)$ represents the shape of the action potential, $H(T)$ is the Heaviside step function and $G(X,X_i;T)$ is the Green's function given by the solution to the following initial value problem:

$$\begin{aligned}
\frac{\partial G}{\partial T}(X,X_i;T) &= \frac{\partial^2 G}{\partial X^2}(X,X_i;T) - G(X,X_i;T) + \delta(X-X_i)\delta(T) \\
G(X,X_i;T) &= \begin{cases} I_A(0,T) & \text{for } X_i = 0 \\ 0 & \text{for } X_i \neq 0 \end{cases}
\end{aligned}$$

and corresponds to the response at time T at position X to a unit impulse at $X = X_i$ and $T = 0$. For a semi-infinite cable with the above nonhomogeneous boundary condition at $X = 0$ the Green's functions $G(X,0;T)$ for $X_i = 0$ and $G(X,X_i;T)$ for $X_i \neq 0$ are given by

$$G(X,0;T) \;=\; \frac{e^{-T}}{\sqrt{4\pi T^3}} X \exp\left(-\frac{X^2}{4T}\right),$$

and

$$G(X,X_i;T) \;=\; \frac{e^{-T}}{\sqrt{4\pi T}}\left[\exp\left(-\frac{(X-X_i)^2}{4T}\right) - \exp\left(-\frac{(X+X_i)^2}{4T}\right)\right],$$

respectively.

*To whom correspondence should be addressed: Email: nicolangelo.iannella@gmail.com

The integral expression for $\Phi_0(X,T)$ can be solved analytically but requires the following integrals to be used,

$$\Gamma(-n-v-1;\frac{X^2}{4T}) = \int_{\frac{X^2}{4T}}^{\infty} z^{-v-2-n}\exp(-z)dz,$$
$$I = \int_0^T \zeta^v \exp\left(\zeta(\alpha-1)-\frac{X^2}{4\zeta}\right)d\zeta,$$

where Γ is the incomplete Gamma function. Keeping only the first two terms of the series, and utilizing the following idenities

$$\Gamma\left(\frac{1}{2};\frac{X^2}{4T}\right) = \sqrt{\pi}\,\mathrm{erfc}\left(\frac{X}{\sqrt{4T}}\right),$$
$$\Gamma\left(-\frac{1}{2};\frac{X^2}{4T}\right) = \frac{4\sqrt{T}}{X}\exp\left(\frac{X^2}{4T}\right)-2\sqrt{\pi}\,\mathrm{erfc}\left(\frac{X}{\sqrt{4T}}\right),$$

leads to the following expression for $\Phi_0(X,T)$ used in the simulations, here the action potential has an after depolarizing tail and is generally given by the following expression,

$$I_A(0,T) = U_0\left(10e^{-AT/7.5}\sin\left((2\pi/150)AT\right)+67e^{-2AT}-70e^{-4AT}+3e^{-AT/24}\right)H(T),$$

where $A=15$ and $H(T)$ is the Heaviside step function. Then, the integral expression for $\Phi_0(X,T)$ can be evaluated analytically (keeping only leading order terms of the expansion) and is given by the following,

$$\begin{aligned}
\Phi_0(X,T) = {} & \frac{10U_0}{U_{\mathrm{peak}}\sqrt{\pi}}\Bigg\{\Bigg(\sin\left(\frac{2\pi AT}{150}\right)\Bigg[1-\left(\frac{X^2}{2}\right)\left(\frac{A}{7.5}-1\right)-\frac{2}{3}\left(\frac{2\pi A}{150}\right)^2\left(\frac{X^2}{4}\right)^2 \\
& +\frac{4}{15}\left(\frac{2\pi A}{150}\right)^2\left(\frac{A}{7.5}-1\right)\left(\frac{X^2}{4}\right)^3\Bigg]+\cos\left(\frac{2\pi AT}{150}\right)\left(\frac{2\pi A}{150}\right)\left(\frac{X^2}{2}\right)\times \\
& \Bigg[1-\frac{2}{3}\left(\frac{A}{7.5}-1\right)\left(\frac{X^2}{4}\right)^2-\frac{2}{45}\left(\frac{2\pi A}{150}\right)^2\left(\frac{X^2}{4}\right)^2 \\
& +\frac{4}{315}\left(\frac{2\pi A}{150}\right)^2\left(\frac{A}{7.5}-1\right)\left(\frac{X^2}{4}\right)^3\Bigg]\Bigg)\pi\mathrm{erfc}\left(\frac{X}{\sqrt{4T}}\right)e^{-AT/7.5} \\
& +\sin\left(\frac{2\pi AT}{150}\right)X\sqrt{T}\Bigg[\left(\frac{A}{7.5}-1\right)-\frac{1}{2}\left(\frac{2\pi AT}{150}\right)^2\left(\frac{T}{3}-\frac{X^2}{6}\right) \\
& -\frac{1}{2}\left(\frac{2\pi AT}{150}\right)^2\left(\frac{A}{7.5}-1\right)\left(\frac{T^2}{5}-\frac{X^2T}{30}+\frac{X^4}{60}\right)\Bigg]\exp\left(-\frac{X^2}{4T}-\frac{AT}{7.5}\right) \\
& -\cos\left(\frac{2\pi AT}{150}\right)X\sqrt{T}\exp\left(-\frac{X^2}{4T}-\frac{AT}{7.5}\right)\Bigg[\frac{2\pi A}{150} \\
& +\left(\frac{2\pi AT}{150}\right)\left(\frac{A}{7.5}-1\right)\left(\frac{T}{3}-\frac{X^2}{6}\right)-\frac{1}{6}\left(\frac{2\pi AT}{150}\right)^3\left(\frac{T^2}{5}-\frac{X^2T}{30}+\frac{X^4}{60}\right) \\
& -\frac{1}{6}\left(\frac{2\pi AT}{150}\right)^3\left(\frac{A}{7.5}-1\right)\left(\frac{T^3}{7}-\frac{X^2T^2}{70}+\frac{X^4T}{420}-\frac{X^6}{840}\right)\Bigg] \\
& +\Bigg[6.7\left(1-\frac{X^2}{2}(2A-1)\right)e^{-2AT}-7\left(1-\frac{X^2}{2}(4A-1)\right)e^{-4AT} \\
& +0.3\left(1-\frac{X^2}{2}\left(\frac{A}{24}-1\right)\right)e^{-AT/24}\Bigg]\sqrt{\pi}\mathrm{erfc}\left(\frac{X}{\sqrt{4T}}\right) \\
& +4X\sqrt{T}\,\exp\left(-\frac{X^2}{4T}\right)\Bigg[6.7\left(\frac{2A-1}{4}\right)e^{-2AT}-7\left(\frac{2A-1}{4}\right)e^{-4AT} \\
& +0.3\left(\frac{(A/24-1)}{4}\right)e^{-AT/24}\Bigg]\Bigg\}.
\end{aligned}$$

where erfc is the complementary error function.

The descriptions of the ionic currents used in the simulations

Sodium current I_{Na}

$$\begin{aligned}
I_{\mathrm{Na}} &= \varepsilon g_{\mathrm{Na}} \mathcal{N}_{\mathrm{Na}}(X_i) m^3 h(\Phi_{\mathrm{Na}} - \Phi) \\
\frac{1}{\tau_m}\frac{\partial m}{\partial T} &= \alpha_m - (\alpha_m + \beta_m) m \\
\alpha_m &= 0.182(\Phi U_{\mathrm{peak}} + Er + 35)/(1 - \exp(-(\Phi U_{\mathrm{peak}} + Er + 35)/9)) \\
\beta_m &= -0.124(\Phi U_{\mathrm{peak}} + Er + 35)/(1 - \exp(\Phi U_{\mathrm{peak}} + Er + 35)/9)
\end{aligned}$$

$$\begin{aligned}
\frac{1}{\tau_m}\frac{\partial h}{\partial T} &= (h_\infty - h)/\tau_h \\
\alpha_h &= 0.024(\Phi U_{\mathrm{peak}} + Er + 50)/(1 - \exp(-(\Phi U_{\mathrm{peak}} + Er + 50)/5)) \\
\beta_h &= -0.0091(\Phi U_{\mathrm{peak}} + Er + 75)/(1 - \exp(\Phi U_{\mathrm{peak}} + Er + 75)/5) \\
h_\infty &= 1/(1 + \exp(\Phi U_{\mathrm{peak}} + 65)/6.2) \\
\tau_h &= 1/(\alpha_h + \beta_h)
\end{aligned}$$

Potassium current I_{K}

$$\begin{aligned}
I_{\mathrm{K}} &= \varepsilon g_{\mathrm{K}} \mathcal{N}_{\mathrm{K}}(X_i) n(\Phi_{\mathrm{K}} - \Phi) \\
\frac{1}{\tau_m}\frac{\partial n}{\partial T} &= \alpha_n - (\alpha_n + \beta_n) n \\
\alpha_n &= 0.02(\Phi U_{\mathrm{peak}} + Er - 20)/(1 - \exp(-(\Phi U_{\mathrm{peak}} + Er - 20)/9)) \\
\beta_n &= -0.002(\Phi U_{\mathrm{peak}} + Er - 20)/(1 - \exp(\Phi U_{\mathrm{peak}} + Er - 20)/9)
\end{aligned}$$

Transient Potassium A-current $I_{\mathrm{K(A)}}$

$$\begin{aligned}
I_{\mathrm{K(A)}} &= \varepsilon g_{\mathrm{K(A)}} \mathcal{N}_{\mathrm{K(A)}}(X_i) m^4 h(\Phi_{\mathrm{K(A)}} - \Phi) \\
\frac{1}{\tau_m}\frac{\partial m}{\partial T} &= (m_\infty - m)/\tau_{m_{\mathrm{K(A)}}} \\
\frac{1}{\tau_m}\frac{\partial h}{\partial T} &= (h_\infty - h)/\tau_{h_{\mathrm{K(A)}}} \\
m_\infty &= 1/(1 + \exp(-(\Phi U_{\mathrm{peak}} + 60)/8.5)) \\
h_\infty &= 1/(1 + \exp((\Phi U_{\mathrm{peak}} + 78)/6) \\
\tau_{m_{\mathrm{K(A)}}} &= 0.185 + 0.5/[\exp((\Phi U_{\mathrm{peak}} + 35.8)/19.7) + \exp(-(\Phi U_{\mathrm{peak}} + 79.7)/12.7)]
\end{aligned}$$

$$\begin{aligned}
\tau_{h_{\mathrm{K(A)}}} &= 0.5/[\exp((\Phi U_{\mathrm{peak}} + 46)/5) \\
&\qquad + \exp(-(\Phi U_{\mathrm{peak}} + 238)/37.5)] \qquad \text{for } \Phi U_{\mathrm{peak}} \leq -63 \\
&= 9.5 \qquad \text{for } \Phi U_{\mathrm{peak}} > -63
\end{aligned}$$

High-Voltage-Activated (HVA) L-type calcium current $I_{\mathrm{Ca(HVA)}}$:

$$\begin{aligned}
I_{\mathrm{Ca(HVA)}} &= \varepsilon g_{\mathrm{Ca(HVA)}} \mathcal{N}_{\mathrm{Ca(HVA)}}(X_i) m^2(\Phi_{\mathrm{Ca(HVA)}} - \Phi) \\
\frac{1}{\tau_m}\frac{\partial m}{\partial T} &= \alpha_m - (\alpha_m + \beta_m) m \\
\alpha_m &= 1.6/(1 - \exp(-0.072(\Phi U_{\mathrm{peak}} + Er - 5)) \\
\beta_m &= -0.02(\Phi U_{\mathrm{peak}} + Er + 8.9)/(1 - \exp(\Phi U_{\mathrm{peak}} + Er + 8.9)/5)
\end{aligned}$$

Low-Voltage-Activated T-type calcium current $I_{\mathrm{Ca(T)}}$

$$\begin{aligned}
I_{\mathrm{Ca(T)}} &= \varepsilon g_{\mathrm{Ca(T)}} \mathcal{N}_{\mathrm{Ca(T)}}(X_i) m^2 h (\Phi_{\mathrm{Ca(T)}} - \Phi) \\
\frac{1}{\tau_m}\frac{\partial m}{\partial T} &= (m_\infty - m)/\tau_{m_{\mathrm{Ca(T)}}} \\
\frac{1}{\tau_m}\frac{\partial h}{\partial T} &= (h_\infty - h)/\tau_{h_{\mathrm{Ca(T)}}}
\end{aligned}$$

Low-Voltage-Activated T-type calcium current $I_{\mathrm{Ca(T)}}$

$$\begin{aligned}
m_\infty &= \frac{1}{(1+\exp(-(\Phi U_{\mathrm{peak}}+56)/6.5))} \\
\tau_{m_{\mathrm{Ca(T)}}} &= 0.204 + \frac{0.333}{\exp((\Phi U_{\mathrm{peak}}-15.8)/18.2)+\exp(-(\Phi U_{\mathrm{peak}}+131)/16.7)} \\
h_\infty &= 1/(1+\exp(-(\Phi U_{\mathrm{peak}}+80)/4)) \\
\tau_{h_{\mathrm{Ca(T)}}} &= \begin{cases} 0.333\exp((\Phi U_{\mathrm{peak}}+466)/66.6) & \Phi U_{\mathrm{peak}} \leq -81 \\ 9.32 + 0.333\exp(-(\Phi U_{\mathrm{peak}}+21)/10.5) & \Phi U_{\mathrm{peak}} \geq -81 \end{cases}
\end{aligned}$$

Single channel conductance used in the simulations were:

$$\begin{aligned}
g_{\mathrm{Na}} &= 18 \text{ pS} \\
g_{\mathrm{K}} &= 20 \text{ pS} \\
g_{\mathrm{K(A)}} &= 6 \text{ pS} \\
g_{\mathrm{Ca(HVA)}} &= 25 \text{ pS} \\
g_{\mathrm{CaT}} &= 8 \text{ pS} \\
g_{\mathrm{NMDA}} &= 50 \text{ pS}
\end{aligned}$$

Simulations were conducted using 10 equally spaced hotspots between $X = 0$ to $X = 0.3$ dimensionless units ($x = 300\ \mu m$) for all ion channels and a single hotspot of NMDA receptors.

The number of channels per hotspot was calculated by finding the total number of channels in a cable of length 300 μm from the channel density $\overline{g}_\mu$ (units of pS/μm^2) and dividing this by the number of hotspots for the specific channel under consideration. The channel densities used were as follows:

$$\begin{aligned}
\overline{g}_{\mathrm{Na}} &= 100 \text{ pS}/\mu\text{m}^2 \\
\overline{g}_{\mathrm{K}} &= 80 \text{ pS}/\mu\text{m}^2 \\
\overline{g}_{\mathrm{K(A)}} &= 50 \text{ pS}/\mu\text{m}^2 \\
\overline{g}_{\mathrm{Ca(HVA)}} &= 40 \text{ pS}/\mu\text{m}^2 \\
\overline{g}_{\mathrm{Ca(T)}} &= 20 \text{ pS}/\mu\text{m}^2
\end{aligned}$$

Finally, the parameter values for the calculation of the internal accumulation and diffusion of calcium used for the chemical cable were given by the following:

$$\begin{aligned}
[\mathrm{Ca}]_{\mathrm{ref}} &= 2 \text{ mM} \\
D_{\mathrm{Ca}} &= 0.23\ \mu\text{m}^2/\text{msec} \\
D_{\mathrm{M}} &= 0.13\ \mu\text{m}^2/\text{msec} \\
P_m &= 2\ \mu\text{m}/\text{msec} \\
\beta &= 10.
\end{aligned}$$

A value of $\varepsilon = 0.095$ was used in all simulations.
